# Preferential epithelial expression of type-1 cannabinoid receptor (CB1R) in the developing canine embryo

**DOI:** 10.1186/s40064-015-1616-0

**Published:** 2015-12-22

**Authors:** Andrea Pirone, Carla Lenzi, Alessandra Coli, Elisabetta Giannessi, Maria Rita Stornelli, Vincenzo Miragliotta

**Affiliations:** Department of Veterinary Sciences, University of Pisa, Viale Delle Piagge 2, 56124 Pisa, Italy

**Keywords:** Cannabinoid receptor 1, Embryo, Dog, Immunohistochemistry

## Abstract

The use of cannabinoid receptor agonists is gaining a strong interest both in human and veterinary medicine. The potential use of cannabimimetic compounds in companion animals was reviewed in 2007 for their role in tissue inflammation and pain. A better knowledge of type-1 cannabinoid receptor (CB1R) expression on the target population may help in risk management in order to prevent unwanted side effects. We used 30-days old canine embryos to describe the distribution of CB1R by means of immunohistochemistry with a commercially available antibody.CB1R immunoreactivity was mainly epithelial and included most structures of central and peripheral nervous system, inner ear, olfactory epithelium and related structures, eye and thyroid. Further investigative research on the role of the endocannabinoid system in the developmental biology field is needed, however, we show that in the canine species we must consider pregnancy as risk condition for developmental abnormalities that may arise upon the use of CB1R receptor agonists.

## Background

Cannabinoid receptor 1 (CB1R) is a G-protein coupled receptor that mediates the effects of Δ9-tetrahydrocannabinol (Δ^9^THC), the most potent psychotropic constituent of *cannabis sativa* (Xie et al. 2012; Razdan 1986). Since its discovery in 1990 (Matsuda et al. 1990), a “peripheral” receptor was also characterised [i.e., type-2 cannabinoid receptor (CB2R)] (Munro et al. 1993); CB1R and CB2R, together with a family of endogenous lipid ligands and the machinery for their biosynthesis and metabolism, are part of the endocannabinoid system (Skaper and Di Marzo 2012).

The role of CB1R and endocannabinoid signalling has been extensively studied in the adult nervous system: N-arachidonoylethanolamide (AEA) and 2-arachidonoylglycerol (2-AG) are the principal natural CB1R agonists able to mediate a retrograde synaptic signalling (Kano et al. 2009) causing inhibition of neurotransmitter release by presynaptic neurons (Elphick and Egertova 2001). Despite the ubiquitous expression of CB1R (Katona 2009), autoradiographic analysis of the brain distribution of (^3^H)CP-55,940 (a potent Δ^9^THC developed by Pfizer Inc., Groton, CT USA) (Herkenham et al. 1991a, 1991b) together with immunocytochemical investigations using different antibodies showed the highest concentrations of cannabinoid binding sites in the basal ganglia and cerebellum (Egertova et al. 1998; Egertova and Elphick 2000; Tsou et al. 1998; Pettit et al. 1998).

Recent research on the role played by the endocannabinoid system in reproduction was mostly focused on gametogenesis (Grimaldi et al. 2013; El-Talatini et al. 2009) and early events leading to the establishment of pregnancy (Melford et al. 2014): it is reported that high levels of CB1R ligands impair reproductive function causing retarded embryo development, fetal loss and pregnancy failure (Paria et al. 2001; Maccarrone et al. 2002; Maccarrone 2009). Also, absence of mediators are known to adversely affect peri-implantation embryonic development as confirmed by studies in CB1R−/− mice (Wang et al. 2004) sanctioning that the CB1R mediated signalling is essential for regular embryo development with any deviation from physiological expression leading to adverse events.

Despite pregnant bitches are unlikely to be exposed to Δ^9^THC, it is possible that CB1R agonists will be used in the future as therapeutic options for treatment of different disorders (Smith et al. 2010). The CB1R/CB2R receptor agonist, Δ9-tetrahydrocannabinol (Δ9-THC; dronabinol; Marinol) and its synthetic analogue, Nabilone (Cesamet), were approved over 25 years ago as medicines for suppressing nausea and vomiting produced by chemotherapy. Sativex, (GW pharmaceuticals), a drug containing both Δ^9^THC and cannabidiol was licensed in 2010 in the UK and Canada for the treatment of spasticity due to multiple sclerosis in humans and was recently approved in several other countries. Targeting the endocannabinoid system with cannabinoid receptor agonists is yet under investigation for several possible additional therapeutic targets (Pertwee 2012).

The potential use of cannabimimetic compounds in companion animals was reviewed in 2007 for their role in tissue inflammation and pain (Re et al. 2007): palmidrol [(palmitoylethanolamide (PEA)], an AEA analogue, resulted in resolution of clinical signs in cats with eosinophilic granuloma (Scarampella et al. 2001). We recently reported CB1R expression in different cell types of normal canine skin (Campora et al. 2012) and increased levels of PEA and other bioactive lipid mediators in canine atopic dermatitis thus supporting the hypothesis of a protective role of these compounds during this inflammatory process (Abramo et al. 2014).

Potential adverse events might be managed if a better understanding of CB1R anatomical pattern is known: for this reason, this study aims to describe the morphological distribution of CB1R in canine embryos by means of immunohistochemistry.

## Results

**Fig. 1:**
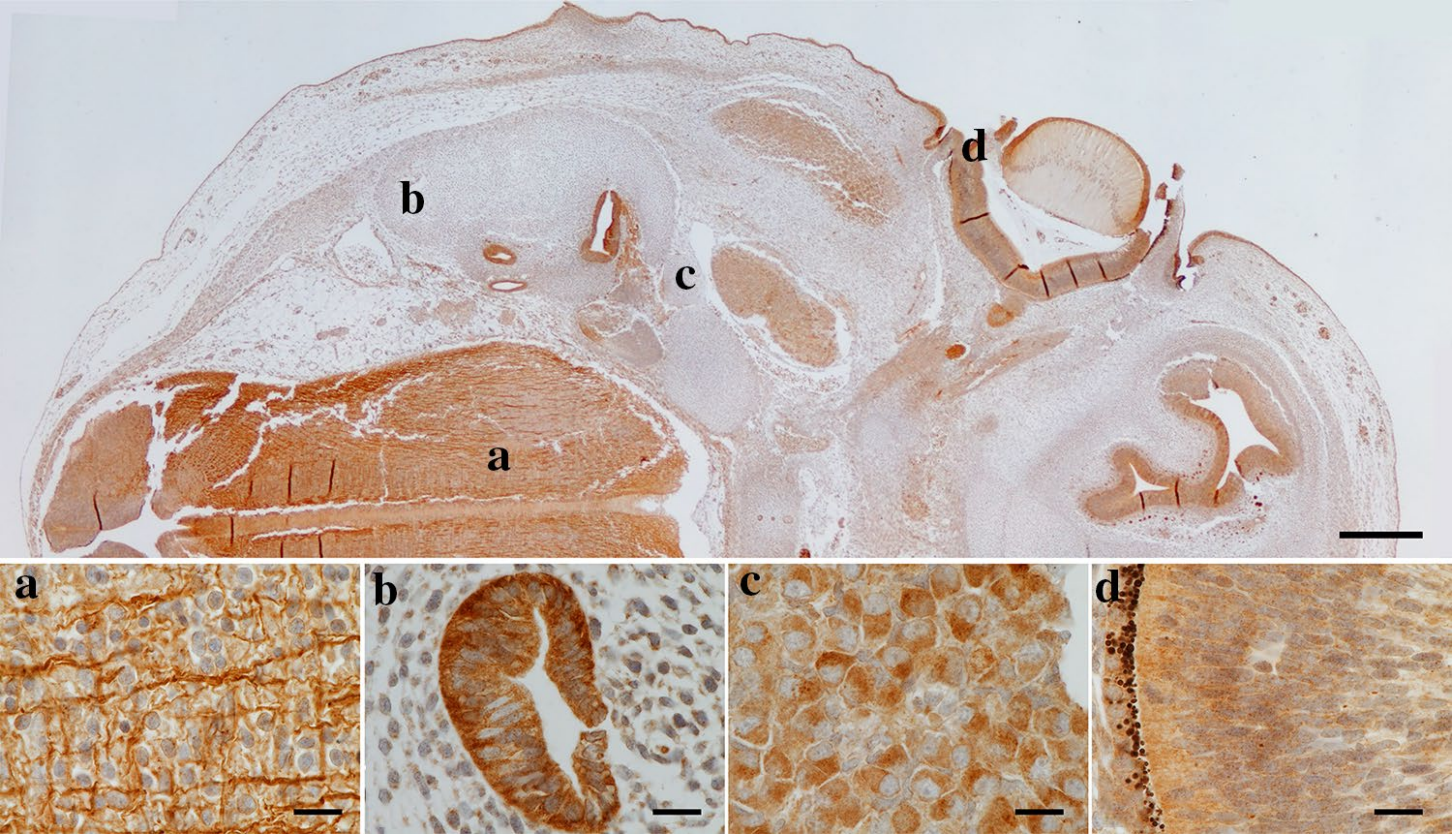
Photomicrographs of the developing canine embryo. The image on top shows all the structures (low magnification) that are enlarged in **a**–**d**; **a** high magnification of the parasagittal area of the hindbrain where rostro-caudal fibres and transverse fibres are visible; **b** high magnification of the developing semi-circular canal (transverse section) where epithelial immunoreactivity is visible as cytoplasmic staining; **c** high magnification of the cochlear ganglion showing cytoplasmic immunostaining of cell bodies; **d** high magnification of the outer layer of the optic cup (future pigmented layer of retina) showing cytoplasmic immunoreactivity. *Scale bar* is 500 µm in the top image and 20 µm in **a**–**d**

Immunoreactivity against CB1R was found in most epithelial structures while the mesenchyme was always found to be devoid of staining (Fig. 1, top image). Strongly stained structures were found in the developing nervous system, sensory organs (primordia of eyes, inner ear related structures and olfactory nerves) and thyroid.

### Nervous system

**Fig. 2:**
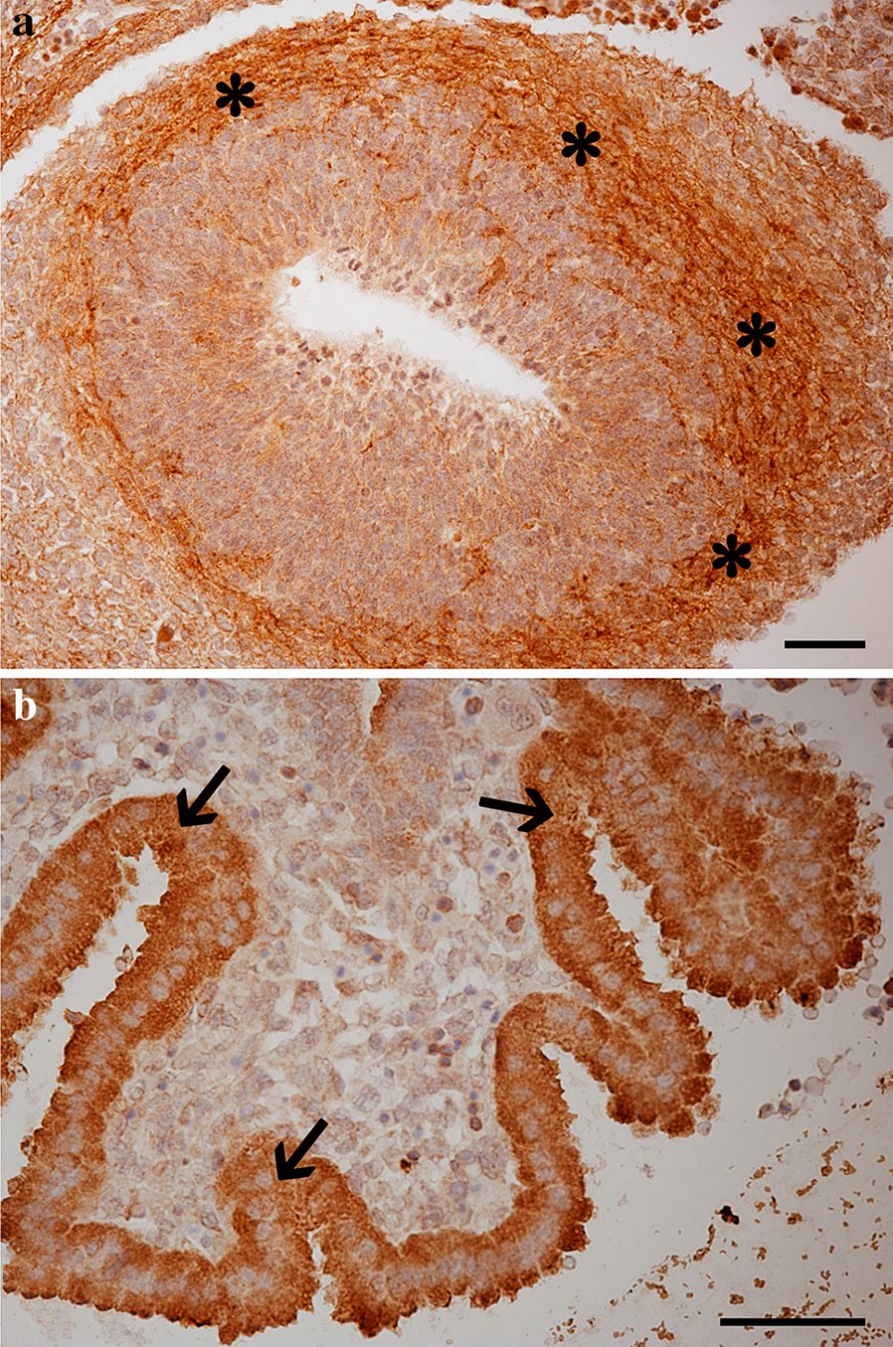
Photomicrographs of the developing central nervous system focusing at the olfactory bulbs (**a**) and choroidal plexa (**b**). **a** *asterisks* indicate positive fibres in the developing olfactory cortex; **b** *arrows* indicate cytoplasmic immunostaining of choroidal plexa epithelial cells. *Scale bars* are equal to 50 µm

**Fig. 3:**
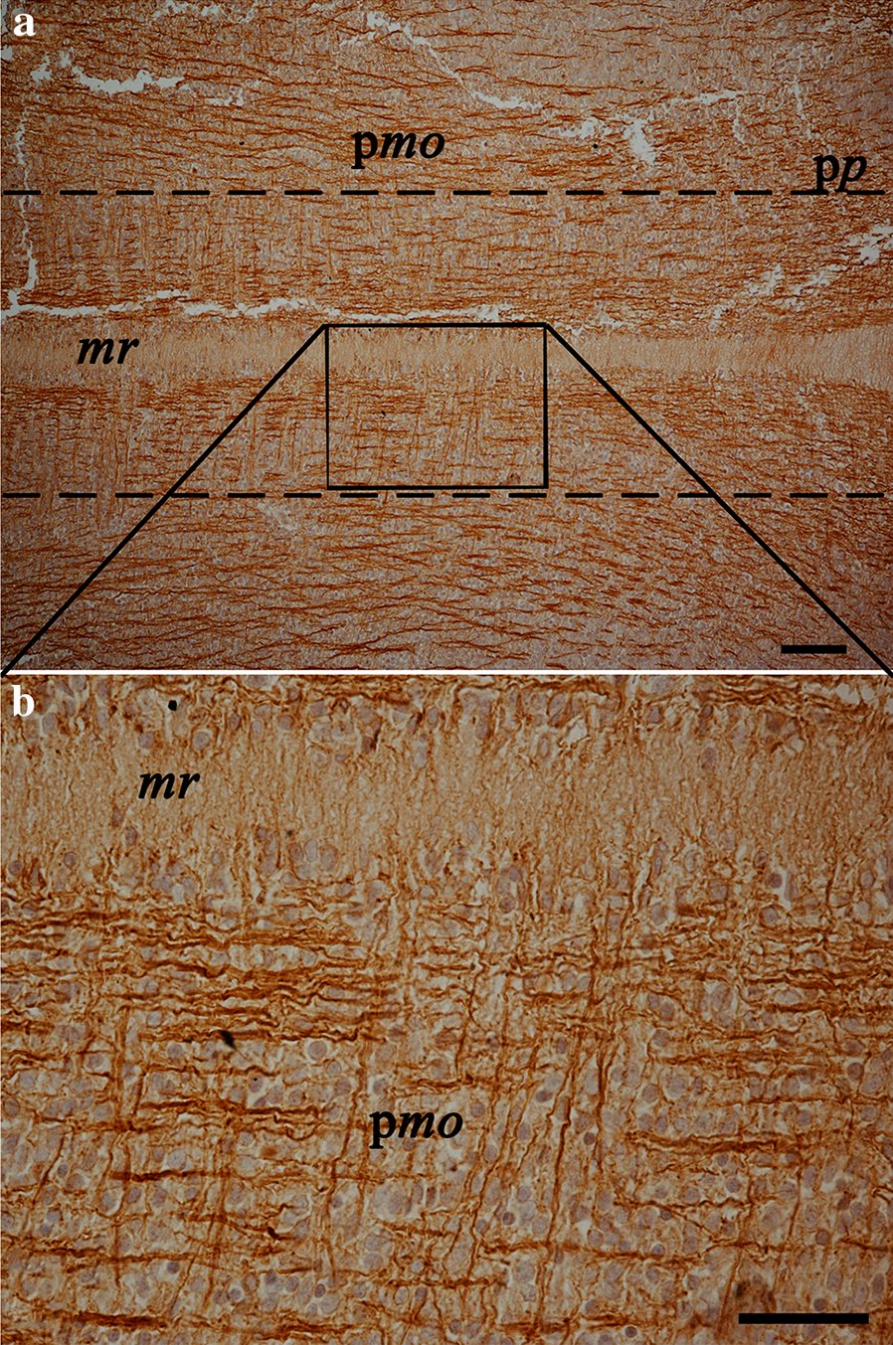
Photomicrographs of the developing central nervous system focusing at the hindbrain; **a** low magnification of primordia of medulla oblongata (p*mo*) and of the pons (p*p*); *mr* indicates medullary raphe; *dashed lines* enclose the parasagittal area where transversal immunoreactive fibres were observed (enlarged in **b**). **b** high magnification of the *square* represented in **a** to show both longitudinal and transversal positive fibres. *Scale bars* are equal to 100 µm (**a**) and 50 µm (**b**)

Numerous CB1R immunoreactive fibers were detected. Some ganglial structures showed cytoplasmic immunoreactivity. The forebrain showed a dense plexus of positive fibres which seemed to arise from the primordia of olfactory bulbs (Fig. 2a). Choroidal plexa also showed the presence of CB1R as cytoplasmic immunostaining (Fig. 2b). Strong immunoreactivity was observed throughout the hindbrain in the section including primordia of medulla oblongata and of the pons. Two sets of fibres were anatomically visible: rostro-caudal fibres and transverse fibres. While rostro-caudal fibres were covering the entire hindbrain (excluding the medullary raphe), transverse fibres were located in the parasagittal area. Fibres crossing the medullary raphe were also visible (Figs. 1a, 3a, b). The developing spinal cord showed CB1R immunoreactivity in its white matter and most of the nerve fibres visible in the embryo body were always stained (not shown).

### Inner ear primordia

**Fig. 4:**
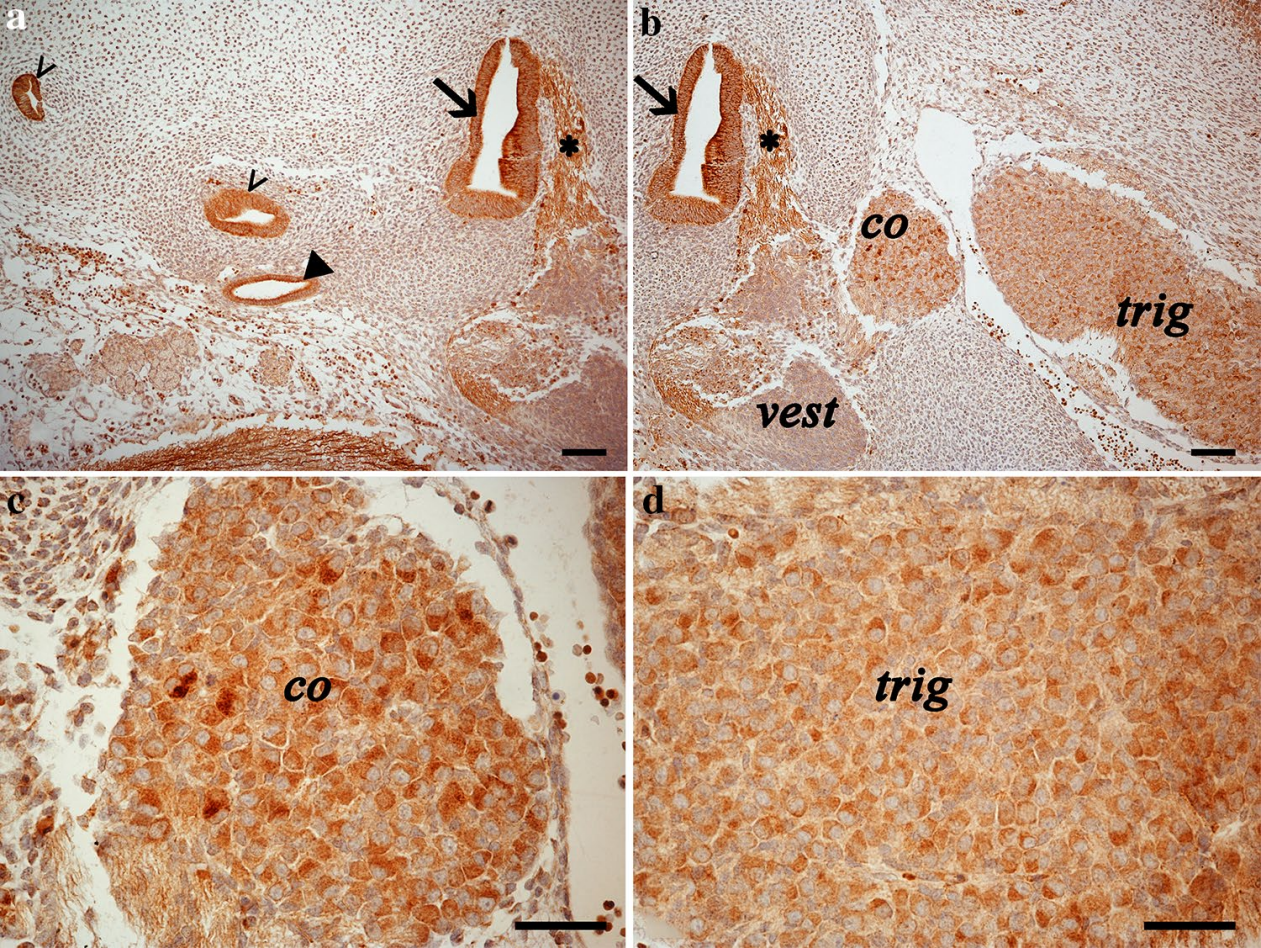
Photomicrographs of the developing inner ear and associated/adjacent nervous structures. *Empty arrowheads* indicate semi-circular canals (**a**, **b**); *Full arrowhead* indicate the endo-lymphatic sac (**a**); *Arrow* indicates the saccular area (**a**, **b**); *Asterisk* indicates the vestibulocochlear nerve immunostained fibres (**a**, **b**). while the trigeminal (*trig*) and the cochlear (*co*) ganglia showed cytoplasmic labelling, the vestibular ganglion (*vest*) was negative to CB1R presence. **c** high magnification of the cochlear (*co*) ganglion; **d** high magnification of the trigeminal (*trig*) ganglion. *Scale bars* are equal to 100 µm in **a**, **b** and to 50 µm in **c**, **d**

Both the lateral and posterior semi-circular canals, the endo-lymphatic sac and the vestibulocochlear nerve showed intense membrane immunoreactivity (Fig. 4a, b). The vestibulocochlear ganglion showed a more intense cytoplasmic staining in its cochlear portion, while the vestibular portion was almost devoid of staining (Fig. 4b, c). The trigeminal ganglion, adjacent to the cochlear one, showed a strong cytoplasmic staining as well (Fig. 4b, d).

### Primordium of the eye

**Fig. 5:**
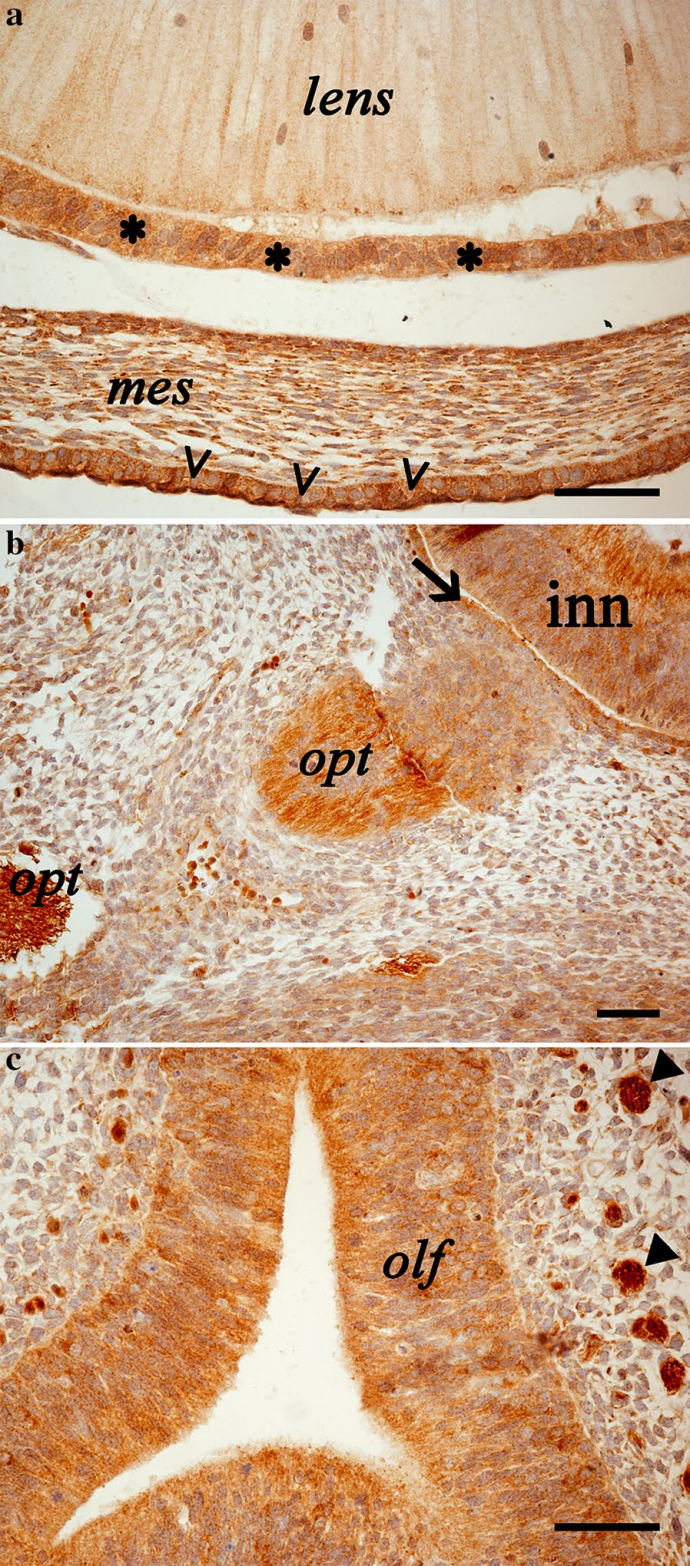
Photomicrographs of the developing eye (**a**, **b**) and nasal cavities. **a** CB1R labelling is visible in the epithelium comprising the capsule of the lens (*asterisks*), in the cell cytoplasm of the mesothelial layer of the cornea (*mes*) and in the surface epithelium of cornea (*arrowheads*); **b** CB1R immunoreactive fibres of the optic nerve (*opt*), the inner (inn) and outer (*arrow*) layer of the optic cup (future nervous and pigmented layer of retina) are shown; **c** CB1R reactivity is visible in the olfactory epithelium (*olf*) and in the olfactory nerve branches (*full arrowheads*). *Scale bars* are equal to 50 µm

Immunoreactivity was observed as cytoplasmic labeling in both the surface epithelium and the mesothelial layer (region of future substantia propria) of cornea, and the cuboidal epithelium covering the anterior part of the lens (Fig. 5a). The inner (neural) layer of the optic cup (future nervous layer of retina) as well the outer layer (future pigment layer of retina) showed CB1R immunostaining in cell cytoplasm (Fig. 5b). The developing optic nerve was strongly marked (Fig. 5b).

### Primordia of the nasal cavities

The olfactory epithelium showed cytoplasmic immunoreactivity; the underlying mesenchyme was devoid of staining but showed intense immunoreactivity of the abundantly represented olfactory nerve branches (Fig. 5c).

### Thyroid

**Fig. 6:**
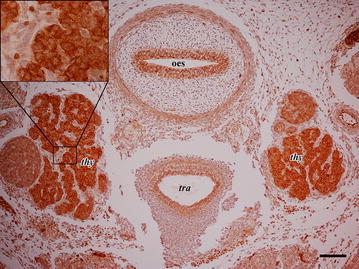
Photomicrographs of the developing thyroid (*thy*). Oesophagus (*oes*), trachea (*tra*) show epithelial staining. *Left* and *right* lobes of thyroid (*thy*) show intense cytoplasm/membrane staining of epithelial cells. Inset is an high magnification to better demonstrate subcellular localization of CB1R immunoreactivity. *Scale bar* is equal to 100 µm

Strong immunoreactivity was observed in the thyroid primordium. Immunostaining was located in cell cytoplasm and membranes (Fig. 6).

## Discussion

Here we report for the first time the immunohistochemical localization of CB1R in the developing canine embryo. In developing rat embryos, CB1R messenger RNA was found in some cells of the neural tube, in several structures of the central and peripheral nervous system, in the retina as well as in two endocrine organs, the thyroid gland and the adrenal gland (Buckley et al. 1998). Our results on CB1R protein expression in canine embryos corroborate some of the above mentioned findings.

Firstly, we found several central and peripheral nervous system structures to express CB1R. Previous studies reported CB1R expression to be spatio-temporally subsequent to neuronal differentiation in the early chick embryo (Begbie et al. 2004); an analogue of Δ^9^THC was then reported to disrupt neural development in chick embryos (Psychoyos et al. 2008). Independent studies concurrently reported that CB1R is required for normal neuronal differentiation, axonal growth and pathfinding during neural development (Watson et al. 2008; Berghuis et al. 2007). A recent study identified SCG10/stathmin-2, a microtubule-binding protein, as the first Δ^9^THC-sensitive molecular effector modulating directional growth of corticofugal axons in the developing cerebrum (Tortoriello et al. 2014).

Then we focused on sensory organs, such as inner ear structures, the developing eye and the olfactory epithelium. Several nervous and non-nervous structures resulted to express CB1R.

Although morphological studies on the role played by endocannabinoids in the auditory system are scarce, it is reported that CB1R mRNA is expressed in the cochlear ganglion of the chick (Stincic and Hyson 2011). This is in accordance with our immunohistochemical data that showed an analogous pattern of staining in the canine embryos.

We showed different structures in the developing eye of canine embryos expressing CB1R. In the same view, Buckley and colleagues found the embryonic rat retina to express CB1R receptor mRNA (Buckley et al. 1998). Further works on the CB1R localization in the eye structures are available in adult rats and humans (Straiker et al. 1999; Yazulla et al. 1999). Taken together these data suggest that the wide distribution CB1R in both the anterior eye and the retina of dogs (present study) and humans (Straiker et al. 1999) may account for an influence of the endocannabinoid system in several different physiological functions of the vision.

With regard to the olfactory epithelium, some data are available in *Xenopus laevis* tadpoles (Czesnik et al. 2007). The mentioned paper shows localization of CB1R-like immunoreactivity on dendrites of olfactory receptor neurons: our data show analogous findings since both the canine embryo olfactory epithelium and the underlying olfactory nerve branches resulted to express the protein.

As stated above, the same work of Buckley et al. showed the thyroid gland and the adrenal gland to express CB1R mRNA in the rat embryo (Buckley et al. 1998). Although we did not find CB1R expression in the developing adrenal glands of canine embryos, thyroid was amongst the major immunostained organs.

Depending on the examined structure, different cellular localization of CB1 were recorded: in particular, while the cytoplasmic compartment was always immunoreactive, we found membrane distribution in the developing thyroid gland. To this regard our study does not allow for mechanistic hypotheses and, although CB1 spatial segregation is likely to affect receptor activity, it is reported that also intracellular CB1 receptors are functional (Rozenfeld and Devi 2008).

## Conclusions

Our work may serve as a basis for further investigative research on the role of the endocannabinoid system in the developmental biology field; also, we show that in the canine specie several developing structures express CB1R. This latter finding should be considered in the use of already available cannabinomimetic compounds. Further investigations are needed to clarify the physiological role of CB1R during canine embryo development.

## Methods

### Animals

Six canine embryos obtained from two different mothers subjected to hysterectomy were used in the present study. Embryos were graded as 30-days-old based on their morphology according to Miller’s Anatomy of the Dog (Evans 1993). Briefly, the entire pregnant uterus was removed during surgery and promptly immersed in 10 % buffered formalin solution. Embryos were dissected after 24 h and kept in fresh 10 % buffered formalin solution for 48 h. Embryos were thus processed for routine paraffin embedding. Serial 5 µm transverse sections were obtained and stained for either morphological (routine Haematoxylin and Eosin staining) or immunohistochemical evaluation of CB1R distribution. Sections were selected in order to include primordia of all the organs present at this embryonic stage.

This research was carried out according to the international regulation on the use of animals for scientific purposes (Directive 2010/63/EU) and all experimental procedures were approved by the local ethical committee.

### Immunohistochemical procedure

Immunohistochemistry was performed on serial sections using a rabbit polyclonal anti-Cannabinoid Receptor I antibody (1:50, abcam, ab23703); a synthetic peptide corresponding to C terminal amino acids 461-472 of Human Cannabinoid Receptor was used to raise the antibody Epitope retrieval was carried out at 120 ℃ in a pressure cooker for 5 min with a Tris/EDTA buffer pH 9.0. Sections were pretreated in 1 % H_2_O_2_ (in 0.1 M PBS, pH 7.4, 10 min) to quench endogenous peroxidase activity, then rinsed with 0.05 % tween-20 detergent (in 0.1 M PBS, 3 × 10 min.), and blocked with 5 % goat normal serum (s-1000, Vector) (in 0.1 M PBS, 1 h). Sections were incubated overnight at 4 °C in a solution containing the rabbit anti-CB1R, 2 % goat normal serum, 0.05 % triton X-100 (in 0.1 M PBS). Subsequently they were rinsed in 0.1 M PBS, (3 × 10 min), followed by 1 h incubation with a biotinylated goat anti-rabbit immunoglobulin (BA-1000, Vector Labs, Burlingame, CA), diluted 1:300 in PBS. Sections were again rinsed in 0.1 M PBS, 3 × 10 min. After 1 h incubation with the ABC complex (pk-7200, Vector, Burlingame, CA) followed by further washes in 0.1 M PBS, (3 x 10 min) staining was visualized by incubating the sections in diaminobenzidine (DAB) (sk-4105, Vector, Burlingame, CA) solution.

Although we had already tested the primary antibody specificity in the canine specie by incubation with the corresponding peptide (Campora et al. 2012), substitution of either the primary antibody, or anti-rabbit IgG, or the ABC complex by PBS or non-immune serum was performed. Under these conditions the immunostaining was abolished.

The microphotographs were taken with a light microscope (Leitz Diaplan, Wetzlar, Germany) connected to a PC via a Nikon digital system (Digital Sight DS-U1, NIS-Elements BR-4.13.00 software).

## Abbreviations

2-AG: 2-arachidonoylglycerol
AEA: N-arachidonoylethanolamide
CB1R: type-1 cannabinoid receptor
CB2R: type-2 cannabinoid receptor
co: cochlear
Δ9-THC: Δ9-tetrahydrocannabinol
mes: mesothelial layer of the cornea
mr: medullary raphe
oes: oesophagus
opt: optic nerve
PEA: palmitoylethanolamide
pmo: primordia of medulla oblongata
pp: pons
thy: developing thyroid
tra: trachea
trig: trigeminal
vest: vestibular
